# Exploring the Sublethal Impacts of Cu and Zn on *Daphnia magna*: a transcriptomic perspective

**DOI:** 10.1186/s12864-024-10701-8

**Published:** 2024-08-19

**Authors:** Berkay Paylar, Yared H. Bezabhe, Jana Jass, Per-Erik Olsson

**Affiliations:** 1 Biology, The Life Science Center, School of Science and Technology, Örebro University, SE-701 82, Örebro, Sweden; 2 Örebro, Sweden

**Keywords:** Gene expression, Transcriptomics, Biomarker

## Abstract

Metal contamination of aquatic environments remains a major concern due to their persistence. The water flea *Daphnia magna* is an important model species for metal toxicity studies and water quality assessment. However, most research has focused on physiological endpoints such as mortality, growth, and reproduction in laboratory settings, as well as neglected toxicogenomic responses. Copper (Cu) and zinc (Zn) are essential trace elements that play crucial roles in many biological processes, including iron metabolism, connective tissue formation, neurotransmitter synthesis, DNA synthesis, and immune function. Excess amounts of these metals result in deviations from homeostasis and may induce toxic responses. In this study, we analyzed *Daphnia magna* transcriptomic responses to IC_5_ levels of Cu (120 µg/L) and Zn (300 µg/L) in environmental water obtained from a pristine lake with adjusted water hardness (150 mg/L CaCO_3_). The study was carried out to gain insights into the Cu and Zn regulated stress response mechanisms in *Daphnia magna* at transcriptome level. A total of 2,688 and 3,080 genes were found to be differentially expressed (DEG) between the control and Cu and the control and Zn, respectively. There were 1,793 differentially expressed genes in common for both Cu and Zn, whereas the number of unique DEGs for Cu and Zn were 895 and 1,287, respectively. Gene ontology and KEGG pathways enrichment were carried out to identify the molecular functions and biological processes affected by metal exposures. In addition to well-known biomarkers, novel targets for metal toxicity screening at the genomic level were identified.

**Supplementary Information:**

The online version contains supplementary material available at 10.1186/s12864-024-10701-8.

## Introduction

*Daphnia magna* is a freshwater crustacean that has been widely used as a model organism in ecotoxicology due to its sensitivity to environmental stressors and ease of handling [1–4]. Copper (Cu) and zinc (Zn) are two heavy metals commonly found in aquatic environments as a result of anthropogenic activity. Cu is involved in numerous biological processes including antioxidant defense, muscle development, and immunological functions [5]. Cu also serves as a cofactor for oxidoreductases, oxygenases, hydroxylases, and transferases [6]. Zn is an integral component of several enzymes and plays a key role in growth, development, and immune functions [7]. Deficiencies in Cu and Zn have been linked to a range of health problems. Cu deficiency may lead to impaired growth, anemia, skeletal abnormalities, and compromised immune function [8]. Insufficient Zn intake has similarly been associated with impaired growth, as well as immune dysfunction, delayed sexual maturation, and impaired cognitive functions [9].

To ensure optimal health it is crucial to maintain a sufficient intake of Cu and Zn. However, excessive intake of these trace elements may also cause adverse effects such as metal toxicity and oxidative stress [10]. *Daphnia magna* has been extensively used to study the effects of these heavy metals in aquatic environments [3]. Exposure to Cu and Zn can induce a range of toxic effects in *Daphnia magna*, including impaired growth, reduced reproductive capacity, and premature death [11]. It is important to note that much of the research related to this topic has been performed using exposure mediums prepared in laboratory settings and has focused on physiological endpoints such as mortality and reproduction while neglecting the toxicogenomic endpoints [4, 12]. The need for transcriptomics in ecotoxicology is becoming increasingly important as traditional methods often fail to provide sufficient information about the mechanisms of toxicity and the effects of environmental stressors on living organisms [3, 13]. Transcriptomics provides a comprehensive view of gene expression changes and can identify key genes and pathways involved in stress responses [14]. Traditional methods of toxicity testing may thus provide information on the physiological and behavioral responses to the stressor, while transcriptomics provides a deeper understanding of the molecular mechanisms underlying these responses [11, 15, 16]. Furthermore, transcriptomics can aid in the identification of novel biomarkers of exposure and effect in aquatic organisms [16, 17]. Biomarkers are measurable responses at the sub-organism level that indicate exposure to stressors [18]. They can be used to monitor the health status of organisms in their natural environment [16, 19, 20]. Novel transcriptomic biomarkers identified from the exposure of *Daphnia magna* to heavy metals are promising tools for monitoring the effects of metal exposure [17, 21]. With this aim, we investigated the transcriptome of *Daphnia magna* exposed to environmental water samples with Cu and Zn in concentrations resulting in 5% immobilization (IC_5_). The use of IC_5_, rather than higher values like IC_10_ or IC_20_, or environmental concentrations of Cu or Zn, was employed to ensure that the effects being studied are subtle and do not cause overt harm to the organisms. This allows for a more nuanced understanding of the organism’s response to these stressors [22]. Studying sublethal exposure transcriptomics offers a better understanding of the molecular mechanisms underlying the effects of environmental stressors, which in turn aids in development of effective strategies for environmental monitoring and risk assessment and identification of safer and more sustainable alternatives to harmful chemicals and pollutants.

## Materials and methods

### *Daphnia* magna culture and exposure

Ephippia from the Daphtoxkit (MicroBioTests Inc., Belgium) were activated by rinsing with tap water and incubation for 72 h in standard freshwater (prepared using 67.75 mg/L NaHCO_3_, 294 mg/L CaCl_2_, 123.25 mg/L MgSO_4_ and 5.75 mg/L KCl) under continuous illumination at a temperature of 22 ± 1** °C**. Newly hatched juveniles (< 24 h) were transferred to the test waters directly after hatching and kept in 16-h light and 8-h dark cycle. *Daphnia magna* was fed a mixture of microalgae (*Raphidocelis subcapitata)* and yeast with adjusted ratios during metal exposure to ensure proper feeding.

Test waters used in the present study were prepared by adjusting the water hardness to 150 mg CaCO_3_/L and the pH to 7 of the water obtained from the pristine Kiimatievanjärvi Lake (67°44′33.2"N, 22°10′49.5"E), Sweden. The concentration of potassium in the exposure waters was set to 3 mg/L with KHCO_3_ to ensure proper maintenance for daphnids. The natural concentration of Cu and Zn in the lake water were 1 μg/L and 0.9 μg/L, respectively (Table 1). For the immobilization assay, Cu concentrations were set to 16, 32, 64, 128, 144, 160, 176, and 256 μg/L using CuCl_2_, whereas Zn concentrations were set to 25, 50, 100, 200, 400, 500, 600, 700 and 800 using ZnCl_2_. Control water without any addition of Cu or Zn was also used. Newly hatched *Daphnia magna* juveniles (< 24 h) were transferred and kept in 8 ml of exposure water in 6 well plates (Sarstedt, Germany). Each well contained 20 animals and four replicates were used for each exposure (total animals = 80). Immobility was screened daily using a light microscope. Daphnids not able to swim after gentle agitation were considered immobile. The percentage of immobilized daphnids at 96 h was plotted against the test concentrations. Slopes and curves were calculated to determine the IC_5_ value with 95% confidence limits (*p* < 0.05) using concentrations transformed to logarithmic values with a base of 10.

**Table 1:** Composition of the water used in this study

Element	Base Water	Control	Cu	Zn
Major component concentration (mg/L)				
Ca	0.3	**22**^a^	**22**	**22**
K	< 0.4	**3**	**3**	**3**
Mg	0.1	**11**	**11**	11
Na	0.4	0.4	0.4	0.4
Minor component concentration (µg/L)				
Al	27.9	27.9	27.9	27.9
As	0.1	0.1	0.1	0.1
Ba	1.9	1.9	1.9	1.9
Cd	0	0	0	0
Co	0	0	0	0
Cr	0	0	0	0
Cu	0.9	0.9	**16 to 256**	0.9
Fe	32.2	32.2	32.2	32.2
Hg	< 0.002	< 0.002	< 0.002	< 0.002
Mn	1.9	1.9	1.9	1.9
Mo	< 0.05	< 0.05	< 0.05	< 0.05
Ni	0.1	0.1	0.1	0.1
P	3.3	3.3	3.3	3.3
Pb	0.1	0.1	0.1	0.1
Si	31.8	31.8	31.8	31.8
Sr	2.6	2.6	2.6	2.6
V	0.1	0.1	0.1	0.1
Zn	1	1	1	**25 to 800**
Total Hardness	1.14 mg/L	**150 mg/L**	**150 mg/L**	**150 mg/L**
Doc	3.72 mg/L	3.72 mg/L	3.72 mg/L	3.72 mg/L
pH	6.8	**7**	**7**	**7**

^a^Experimentally adjusted water criteria for exposure is shown in bold

For the transcriptomics assay daphnids were exposed to approximate IC_5_ concentrations of 120 μg Cu/L and 300 μg Zn/L, along with control water. For each treatment, eight replicates containing a total of 35 daphnids were used for RNA extraction and subsequent transcriptomics analysis. In our earlier research [11], we noted that the onset of gene expression related to reproduction occurs around 96-h post-hatching (hph). This time point signifies the transition from the juvenile to the adult phase. Given the significance of reproduction in terms of physiology and its crucial role in ensuring the survival of a population, we aimed to gain insight into this vital physiological process. Following 96 h exposure, daphnids were collected and snap-frozen using liquid nitrogen and stored at -80 °C until RNA extraction.

## RNA isolation for transcriptomics and qRT-PCR

*Daphnia magna* was lysed using 350 μL of TRI Reagent (Sigma) using tissue homogenizer (Precellys Evolution, Bertin Technologies, USA) and RNA extraction was performed using Direct-zol Kit (Zymo Research, USA) according to the manufacturer's instructions. A DeNovix DS-11 spectrophotometer (Wilmington DE, USA) was used to measure RNA concentration and purity (Table S1). cDNA libraries were generated from 100 ng of RNA. Library preparation was carried out by purification of poly-A containing mRNA, mRNA fragmentation, random primed cDNA synthesis, adapter ligation, and adapter specific PCR amplification by INVIEW Transcriptome Discover (Eurofins Genomics, Germany).

## RNA sequencing and transcriptomics analysis

Illumina paired-end read sequencing was carried out to generate 2 × 150 bp reads. Reads were trimmed from three prime ends based on quality score. Average Phred score for the reads were determined for all replicates (Table 2). Reads were aligned to the *Daphnia magna* genome (daphmag2.4) using Hisat2 Aligner followed by quantification to alignment model using Partek genomic software (Partek Inc., St. Louis, USA). Data were normalized by counts per million (CPM) + 1.0E^−4^. For the gene expression analysis, we used DESeq2 [23]. This method allows us to identify genes that show statistically significant differences in expression levels between the groups. It uses a model based on the negative binomial distribution to test for differential expression in digital gene expression data. DESeq2 was chosen as it provides statistical routines for determining differential expression in digital gene expression data using a model based on the negative binomial distribution. This makes it highly suitable for high-throughput count data from RNA-Seq. FDR step-up value was used for follow-up multiple test correction. Transcripts with p-value ≤ 0.05 and FDR step-up value ≤ 0.05 were considered differentially expressed. A BLAST search was executed on the *Daphnia magna* genome, focusing on uncharacterized proteins that were among the top ten for fold change, both upregulated and downregulated using the default parameters. The selection of significant hits was based on e-values, with a focus on those with the highest identity and query coverage. After the initial transcriptomics analysis, Partek’s biomarker computing function was used to identify exposure-specific biomarker genes and to design primers for biomarker validation. Bubble plots were created to highlight GO annotations and KEGG pathway analysis for each exposure by employing identified DEGs using online data analysis and visualization platform (https://www.bioinformatics.com.cn/en).

**Table 2:** Samples were aligned to *Daphnia magna* reference genome by HISAT 2 algorithm and post alignment quality check for the read pairs were generated

Sample	Total Reads	Total Alignments	Aligned	Avg. Coverage Depth	Avg. Length	Avg. Phred	%GC
Con1	40,306,852	68,493,713	88.47%	235.05	150.9	35.71	44.88%
Con2	29,378,208	52,376,206	92.93%	180.4	150.9	35.81	44.73%
Con3	30,431,151	53,777,378	92.24%	186.98	150.9	35.77	44.16%
Con4	23,854,778	42,079,248	92.14%	152.42	150.9	35.76	43.91%
Cu1	26,173,958	44,396,080	90.15%	159.06	150.9	35.3	45.63%
Cu2	34,763,187	61,285,600	91.64%	214.07	150.9	35.82	44.33%
Cu3	41,105,882	73,513,072	93.44%	257.2	150.9	35.72	44.41%
Cu4	32,939,050	58,030,470	91.86%	209.16	150.9	35.76	44.21%
Zn1	46,371,045	81,381,688	90.88%	258.35	150.9	35.82	44.39%
Zn2	30,832,628	54,253,735	92.14%	190.92	150.9	35.73	45.58%
Zn3	38,858,123	69,113,355	92.65%	232.62	150.9	35.81	44.32%
Zn4	31,518,862	54,148,436	89.43%	191.31	150.9	35.72	44.59%

## qPCR validation

cDNA was synthesized using the qScript cDNA synthesis kit (Quanta Biosciences, USA) and 1000 ng of RNA obtained from 4 additional biological replicates, according to the manufacturer's instructions. The primer sequences of genes used for validation are shown in Table S2. qRT-PCR was performed to quantify the expression of the genes using qPCRBIO SyGreenMix Lo-ROX (PCR Biosystems, USA) using the CFX384 Real time PCR detection system (Bio-Rad, USA) with thermal cycling profiles of initial denaturation step at 95 °C for 2 min followed by 35 cycles of 95 °C for 5 s and 60 °C for 30 s. Expression ratios were calculated based on the ΔΔCt method [24]. Four different housekeeping genes were tested (*actin*, *tubulin*, *elong*, *gapdh*) and, ultimately, *gapdh* was selected based on the stability among the treatment groups.

## Statistical analysis

All statistical analyses were performed using GraphPad Prism 8.0.2 software (GraphPad Software, USA) using one-way ANOVA followed by Dunnett’s post-test for multiple group comparison. Statistically significant differences were considered when *p*-values were < 0.05 (*p < 0.05, **p < 0.01 and ***p < 0.001). Principal component analysis (PCA) was used to analyze multivariate data using the SIMCA software, v13.0.3 (Umetrics, Sweden). A number of significant components were validated using cross validation rules. Tolerance ellipse based on Hotelling's T2 was used to check for outliers (95%). For enrichment analysis, terms and pathways with p-values less than 0.05 are considered significant.

## Results

### Determination of IC_5_ levels

**Fig. 1:**
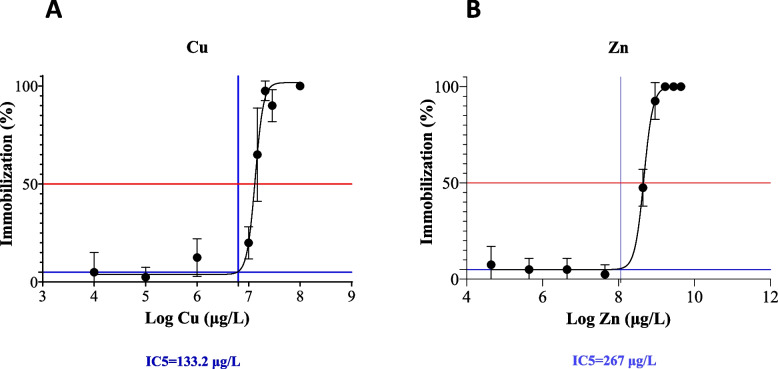
Acute toxicity determination. Twenty *Daphnia magna* neonates (< 24 h old) per well were exposed to varying (**A**) Cu and (**B**) Zn concentrations for 96 h and the survival rate was recorded at the end of the exposure. Dose–response curves were generated to calculate IC_5_ values. Mean ± SEM. *N* = 4

The results of the 96 h immobilization assay are presented in Fig. 1, along with the calculated IC_5_ values for Cu (Fig. 1A) and Zn (Fig. 1B). The IC_5_ values for Cu and Zn were determined to be 133 μg/L and 267 μg/L, respectively. These values represent the concentrations of Cu and Zn that would result in a 5% immobilization of the test organisms. The slopes and curves used to derive the IC_5_ values provide an accurate representation of the dose–response relationship between the concentrations of Cu and Zn and the observed level of immobilization. Following the immobilization test, *Daphnia magna* neonates were treated with IC_5_ concentrations (130 μg/L Cu and 300 μg/L Zn) as well as control water with parameters shown in Table 1 for transcriptomics analysis.

## DEG identification

**Fig. 2:**
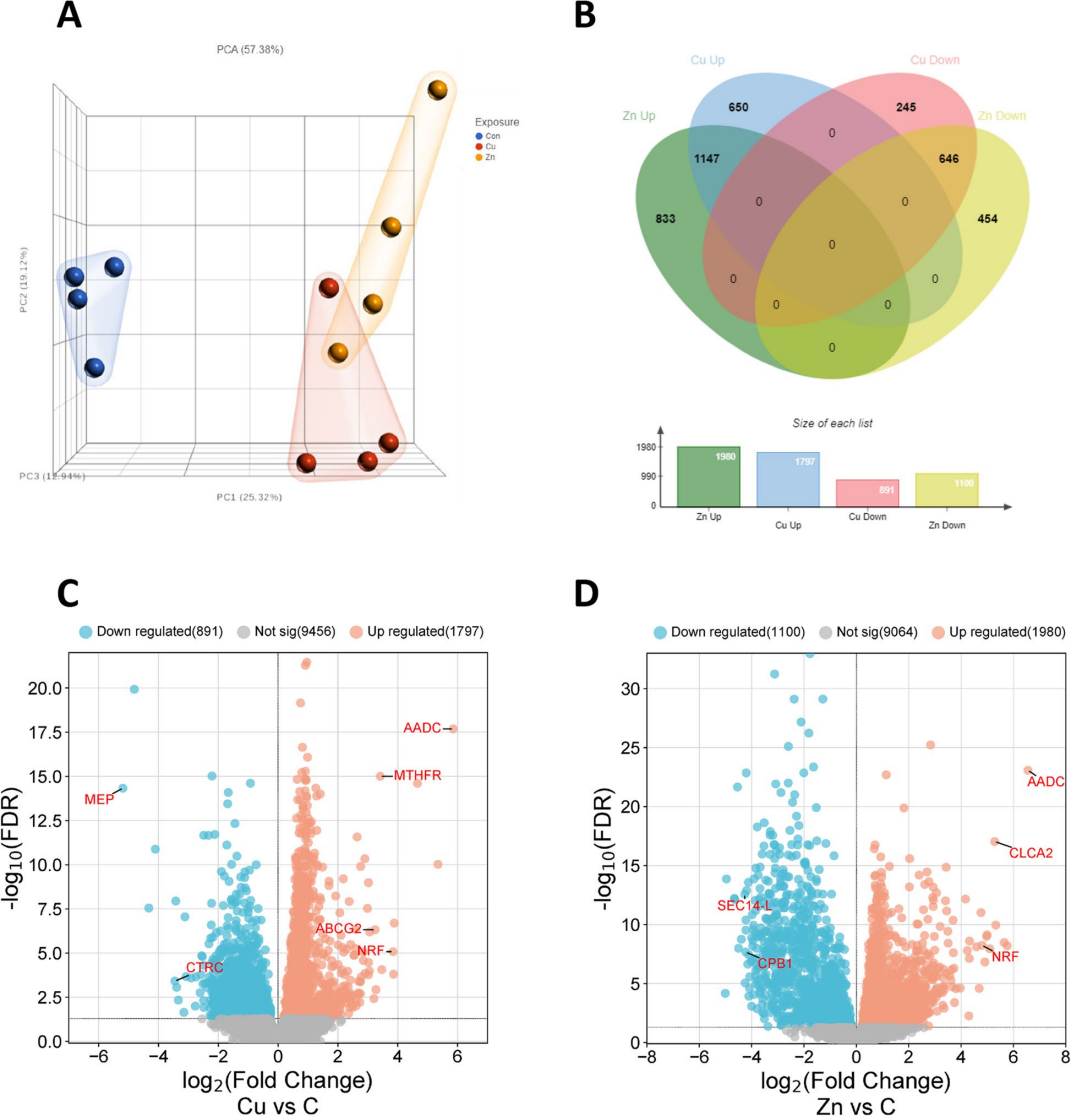
Transcriptomics analysis. **A** Principal component analysis (PCA) was conducted using transcripts with a Lowest Average Coverage (LAC) greater than one. The first component (PC1) accounted for 25.32% of the variance and distinctly segregated the control and exposure groups. **B** Venn diagram illustrates the differentially expressed genes (DEGs) between the control and exposure groups, with a significance level of *p* < 0.05 and a false discovery rate (FDR) less than 0.05. **C** and **D** Volcano plots were created to visualize the effects of Cu and Zn exposures

On average, 33 million reads were generated per sample and over 90 percent of the reads were aligned to *Daphnia* genome with a read quality of 35.73 Phred quality score (Table 2) reflecting the high accuracy of base calling. Principal component analysis displayed variance between the control and exposure groups. PCA explained 57.38% with three components (Fig. 2A). Control and exposure groups were separated clearly with the first principal component, whereas variance between the Cu and Zn exposed samples were less clear and mostly occurred on the secondary component axis.

A total of 12,213 transcripts were identified with a lowest average coverage value greater than one and were used for DEG analysis. The Venn diagram was used to compare the DEGs identified between Cu and Zn exposures (Fig. 2B). There were 1,793 differentially expressed transcripts common to both Cu and Zn exposures. Although 1147 and 646 DEGs were identified for upregulation and downregulation, respectively, we did not identify any DEGs for either Cu or Zn that showed opposite expression (i.e., upregulated in one and downregulated in other, or vice versa). The Volcano plots visualize DEG distribution for both exposure groups (Fig. 2C, 2D) highlighting highly regulated annotated genes. Following the DEG analysis, genes were ranked based on their fold change to identify highly affected transcripts between control and exposure groups. The top ten transcripts for both upregulation and downregulation following Cu and Zn exposure are listed in Table 3 and Table 4, respectively. Uncharacterized proteins were blasted against the *Daphnia magna* genome at default settings and hit with significant e-values and highest identity and query coverage to identify the unknown proteins [25]. Aromatic-L-amino-acid decarboxylase (*aadc*) was the most upregulated gene for both Cu (57.85-fold) and Zn (93.9-fold) exposure. Additionally, complement factor D (*cfd*), putative Nose resistant to fluoxetine protein (*nrf*), leukocyte receptor cluster member 9 (*leng9*), and coagulation factor IX (*fix*) were identified in both exposure settings, as well as an unidentified protein with the locus tag of APZ42_015569. There was only one common transcript (APZ42_014255) among the top ten downregulated genes for both exposures (Cu -8.87-fold, Zn -31.21-fold), coding for an unidentified protein product. Identification of uniquely expressed genes following either Cu or Zn exposure revealed 650 and 833 upregulated as well as 245 and 454 downregulated genes for Cu and Zn, respectively.

**Table 3:** Top ten significantly upregulated and downregulated genes following Cu exposure ranked based on fold change and false discovery rate (FDR) from transcriptomics analysis

Rank	Locus Tag	Gene	*P*-value	FDR	Fold Change
Upregulated					
1	APZ42_026940	Aromatic-L-amino-acid decarboxylase	8.63E-22	2.05E-18	57.85
2	APZ42_018009	Uncharacterized protein (complement factor D)	7.24E-13	9.57E-11	40.59
3	APZ42_018563	Uncharacterized protein	3.48E-18	2.47E-15	25.22
4	APZ42_018715	Uncharacterized protein (proteoglycan 4)	4.72E-09	2.03E-07	14.74
5	APZ42_015569	Uncharacterized protein	9.71E-06	1.57E-04	14.50
6	APZ42_013961	putative Nose resistant to fluoxetine protein	3.36E-07	8.35E-06	14.33
7	APZ42_028101	Uncharacterized protein (leukocyte receptor cluster member 9)	4.85E-06	8.62E-05	11.06
8	APZ42_019649	Methylenetetrahydrofolate reductase	9.16E-19	9.92E-16	10.66
9	APZ42_014001	Uncharacterized protein (coagulation factor IX isoform X1)	1.09E-04	1.18E-03	9.66
10	APZ42_026114	ATP-binding cassette sub-family G member 2	1.24E-08	4.74E-07	9.46
Downregulated					
1	APZ42_024317	Metalloendopeptidase-like protein	7.47E-18	4.68E-15	-36.51
2	APZ42_021089	Uncharacterized protein (mucin-5AC)	2.98E-24	1.18E-20	-28.00
3	APZ42_021101	Uncharacterized protein	5.10E-10	2.81E-08	-20.00
4	APZ42_023964	Uncharacterized protein (procathepsin L isoform X2)	7.14E-14	1.31E-11	-17.20
5	APZ42_018880	Chymotrypsin-C	2.79E-05	3.83E-04	-10.93
6	APZ42_032826	Pancreatic lipase-related protein 2	1.82E-10	1.14E-08	-10.72
7	APZ42_033052	Uncharacterized protein	7.63E-05	8.77E-04	-10.48
8	APZ42_019741	Jonah 65Aiv	5.67E-04	4.57E-03	-10.09
9	APZ42_014255	Uncharacterized protein	4.07E-03	2.00E-02	-8.87
10	APZ42_018190	Uncharacterized protein	1.83E-09	8.88E-08	-8.67

**Table 4:** Top ten significantly upregulated and downregulated genes following Zn exposure ranked based on fold change and false discovery rate (FDR) from transcriptomics analysis

Rank	Locus Tag	Gene	*P*-value	FDR	Fold Change
Upregulated					
1	APZ42_026940	Aromatic-L-amino-acid decarboxylase	6.84E-27	8.30E-24	93.8
2	APZ42_012256	Uncharacterized protein	1.97E-10	6.28E-09	54.22
3	APZ42_015569	Uncharacterized protein	9.65E-11	3.31E-09	50
4	APZ42_028101	Uncharacterized protein (leukocyte receptor cluster member 9)	2.07E-12	1.09E-10	40.06
5	APZ42_022978	Calcium-activated chloride channel regulator 2	2.41E-20	9.14E-18	38.88
6	APZ42_017242	Uncharacterized protein	3.68E-10	1.11E-08	33.98
7	APZ42_018217	Uncharacterized protein	1.72E-11	7.34E-10	31.77
8	APZ42_018009	Uncharacterized protein (complement factor D)	2.46E-11	1.00E-09	31.4
9	APZ42_014001	Uncharacterized protein (coagulation factor IX isoform X1)	6.50E-09	1.48E-07	29.94
10	APZ42_013961	putative Nose resistant to fluoxetine protein	1.76E-10	5.68E-09	27.86
Downregulated					
1	APZ42_014255	Uncharacterized protein	5.89E-06	6.73E-05	-32.21
2	APZ42_018541	Uncharacterized protein	9.02E-17	1.36E-14	-31.21
3	APZ42_021312	Uncharacterized protein (proline-rich extensin-like protein EPR1)	6.46E-15	5.90E-13	-25.27
4	APZ42_024011	Uncharacterized protein (ervatamin-B-like)	8.44E-15	7.37E-13	-25.11
5	APZ42_015784	Uncharacterized protein	2.86E-25	2.17E-22	-23.31
6	APZ42_022638	Uncharacterized protein (collagen alpha-1(I) chain)	3.33E-10	1.01E-08	-22.64
7	APZ42_032551	Uncharacterized protein (glycine, alanine and asparagine-rich protein)	4.69E-11	1.75E-09	-21.04
8	APZ42_015801	SEC14 4-like protein	2.57E-15	2.60E-13	-19.21
9	APZ42_017574	Uncharacterized protein (endochitinase A)	6.04E-07	8.81E-06	-19
10	APZ42_012198	putative Carboxypeptidase B	7.81E-10	2.16E-08	-18.71

## Biomarker selection and qPCR validation

**Fig. 3:**
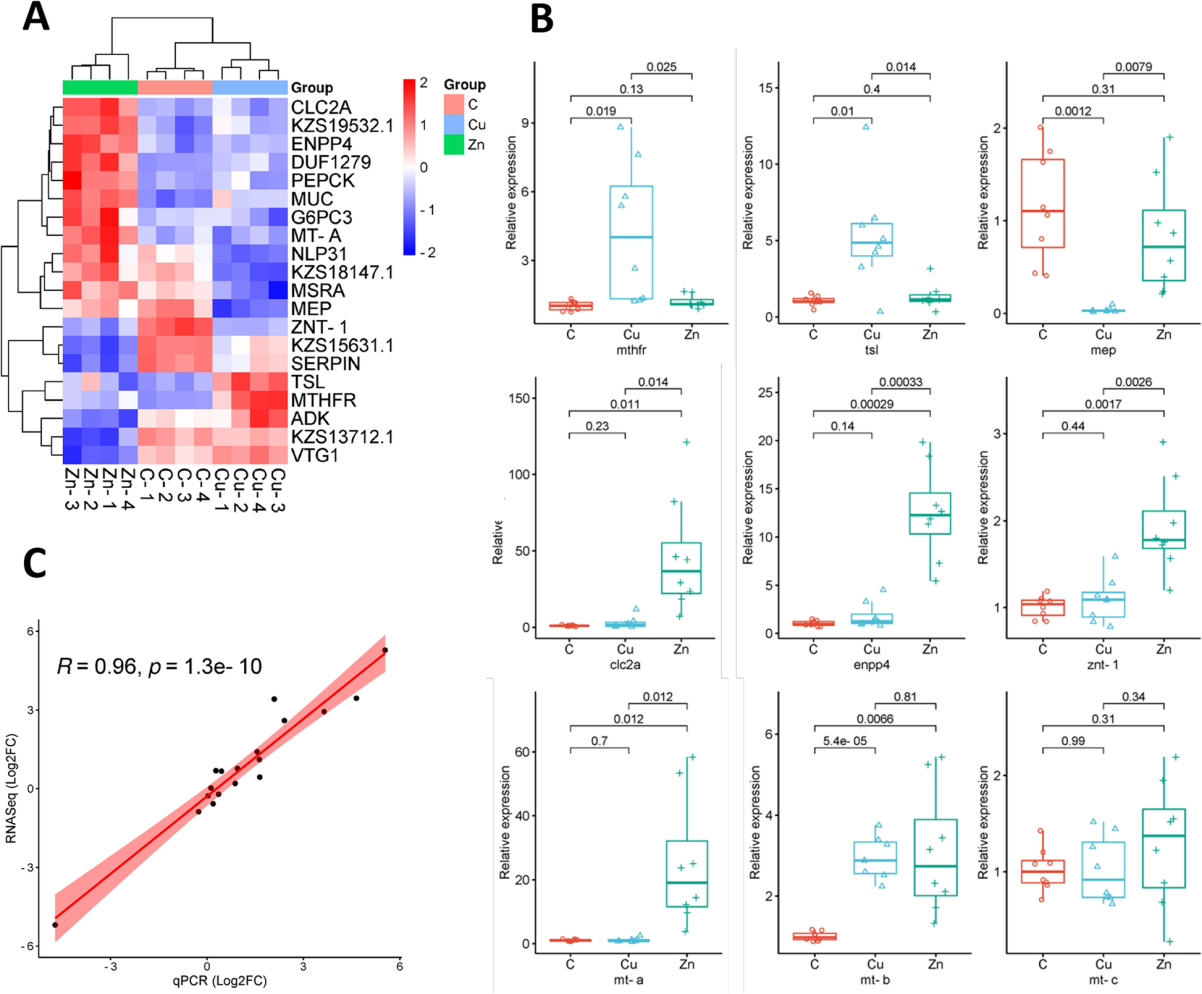
Biomarker identification. **A** Heatmap and hierarchical clustering of potential biomarkers was generated using top ranked genes in biomarker analysis using Partek. **B** qPCR was carried out to to validate potential biomarker genes (Mean ± SEM. *N* = 8). **C** Linearity between RNA-Seq and qPCR reseults were analyzed using Pearson correlation coefficient

Using the Partek biomarker-computing function, a hierarchical cluster and a heatmap with unique biomarker genes for both Cu and Zn exposures was created (Fig. 3A). In addition to *metallothionein* (*mt*) homologs (known for their role in metal sequestration), a subset of the identified potential biomarkers underwent qPCR validation using a different set of biological replicates (Fig. 3B). All potential biomarkers were subsequently validated using qPCR, affirming their status as both unique and significant DEGs for their respective exposure groups. The *Daphnia magna mt* genes differed in their metal responses, with *mt-a* being upregulated by Zn exposure, whereas *mt-b* was upregulated by both Cu and Zn exposure, and *mt-c* remained unaffected by either metal exposure. The gene expression levels following Zn exposure were higher for *mt-a* (25-fold) as opposed to *mt-b* (threefold). The copy numbers obtained from transcriptomics and the relative expression results from qPCR were compared using Pearson's correlation analysis (Fig. 3C). An R-value of 0.96 and significant p-values indicate a strong correlation between the transcriptomics copy numbers and the qPCR relative expression results.

## Gene set enrichment

**Fig. 4:**
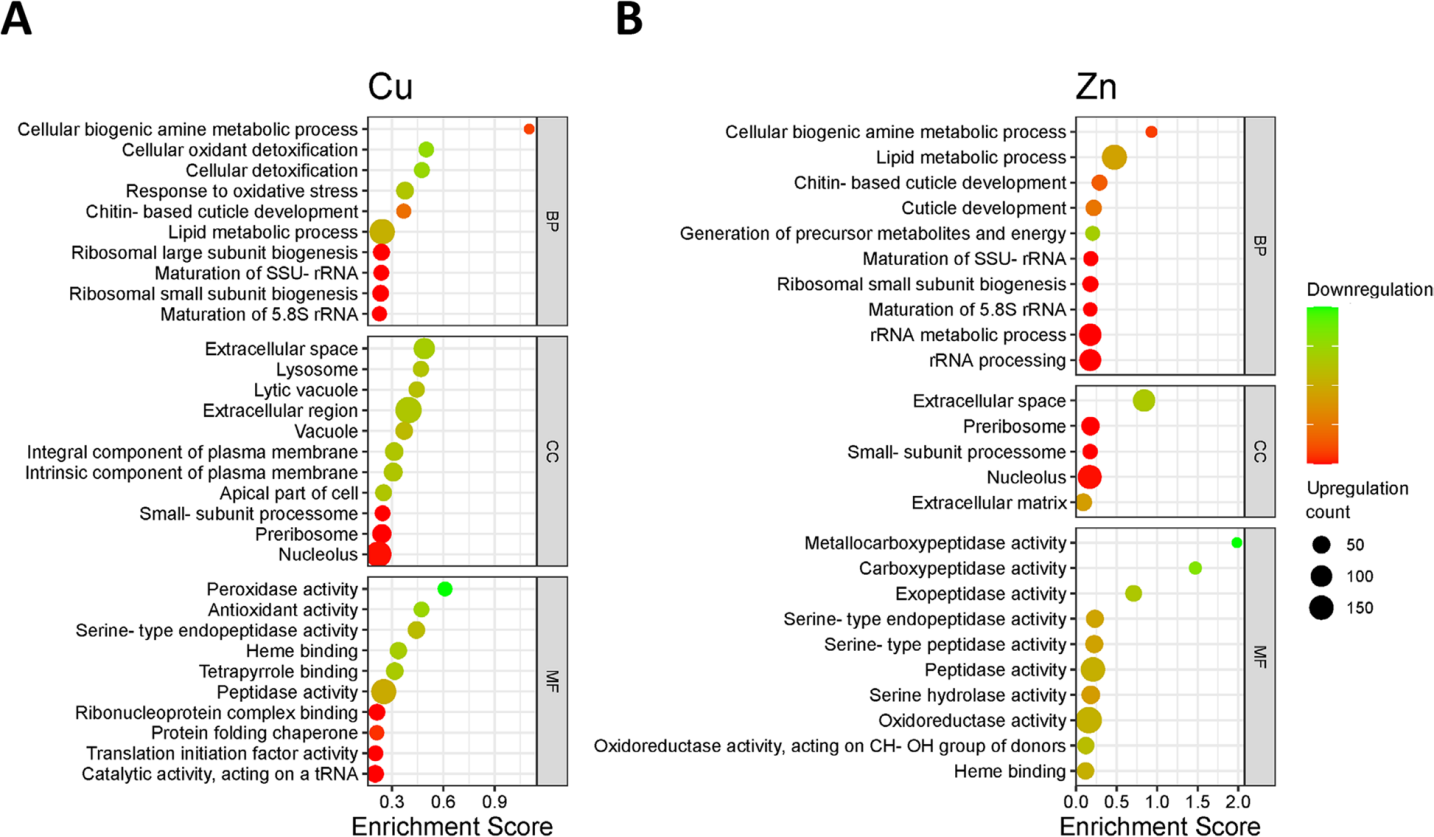
Functional annotation of the Gene Ontology (GO). Enrichment was performed using Differentially Expressed Genes (DEGs) for each exposure. To prioritize the GO terms, an enrichment score was applied. The size of the dots in the visualization corresponds to the number of genes associated with the respective terms. The color scheme applied to these dots reflects the percentage of genes upregulated or downregulated within the specified GO term

We conducted a functional enrichment analysis on the differentially expressed genes within their respective groups. The analysis was performed using the STRING database [26]. The aim was to understand the impact of Cu and Zn exposure on different biological processes, molecular functions, and cellular components (Fig. 4A, Fig. 4B, and Table 5).

**Table 5:** Biological pathways in *Daphnia magna* enriched by upregulated genes after copper and zinc exposure. Related terms were summarized by the top- and lowest-level pathways/events

Metal exposure	Affected cellular process/pathway	Low-level pathway/event term
Zinc	Metabolism of RNA	rRNA processing in the nucleus and cytosol
		Major pathway of rRNA processing in the nucleolus and cytosol
	Transport of small molecules	Stimuli-sensing channels
Copper	Cell Cycle	Ubiquitin Mediated Degradation of Phosphorylated Cdc25A
		Separation of Sister Chromatids
		Cyclin E associated events during G1/S transition
		FBXL7 down-regulates AURKA during mitotic entry and in early mitosis
		Cdc20:Phospho-APC/C mediated degradation of Cyclin A
		APC/C:Cdc20 mediated degradation of Securin
		SCF(Skp2)-mediated degradation of p27/p21
	DNA Replication	Assembly of the pre-replicative complex
		Orc1 removal from chromatin
		CDK-mediated phosphorylation and removal of Cdc6
	Cellular responses to stimuli	GSK3B and BTRC:CUL1-mediated-degradation of NFE2L2
		Regulation of HSF1-mediated heat shock response
		Oxygen-dependent proline hydroxylation of Hypoxia-inducible Factor Alpha
	Metabolism of proteins	UCH proteinases
		SUMO E3 ligases SUMOylate target proteins
	Metabolism of RNA	Major pathway of rRNA processing in the nucleolus and cytosol
		AUF1 (hnRNP D0) binds and destabilizes mRNA
	Gene expression (Transcription)	RUNX1 regulates transcription of genes involved in differentiation of HSCs
		Regulation of RUNX2 expression and activity
		Regulation of RUNX3 expression and activity
	Immune system	Activation of NF-kappaB in B cells
		Cross-presentation of soluble exogenous antigens (endosomes)
		Downstream TCR signaling
		NIK– > noncanonical NF-kB signaling
		Interleukin-1 signaling
		Dectin-1 mediated noncanonical NF-kB signaling
		FCERI mediated NF-kB activation
	Metabolism	Regulation of ornithine decarboxylase (ODC)
	Signal transduction	Regulation of RAS by GAPs
		Degradation of GLI1 by the proteasome
		GLI3 is processed to GLI3R by the proteasome
		Degradation of AXIN
		Degradation of DVL
		Asymmetric localization of PCP proteins

**Fig. 5:**
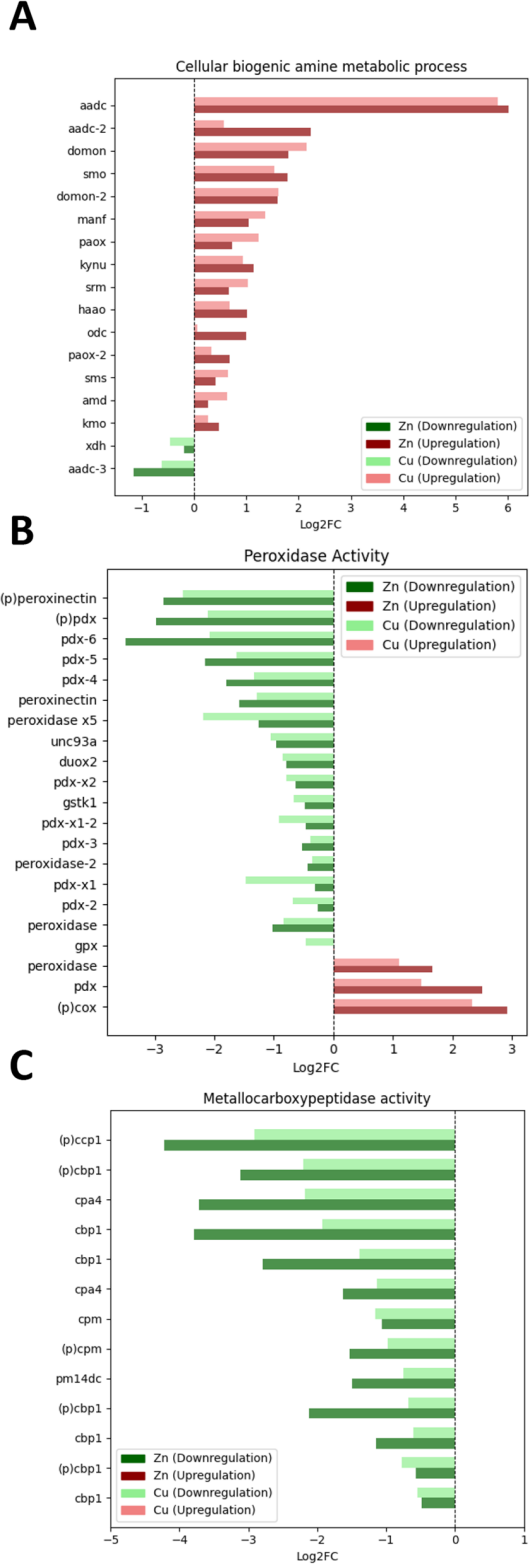
Expression level of genes in highest enriched pathways. **A** Cellular biogenic amine process was the most enriched biological function for both exposures. **B** Peroxidase activity and (**C**) Metallocarboxypeptidase activity were also further identified as the most enriched molecular functions for Cu and Zn, respectively

The cellular biogenic amine metabolic process emerged as the most enriched Gene Ontology (GO) term in the biological process category for both exposures. A significant proportion of the genes associated with this term exhibited upregulation for both exposures (Fig. 5A). Cu exposure led to significant enrichment in molecular processes such as cellular oxidant detoxification, response to oxidative stress, and lipid metabolic process. Similarly, Zn exposure influenced biological processes such as lipid metabolic process and cuticle development. Both Cu and Zn exposures significantly upregulated ribosomal subunit biogenesis, underscoring their importance in cellular homeostasis. Cellular compartments such as the extracellular space, lysosome, and extracellular region were significantly influenced by Cu exposure, with genes related to the extracellular space showing noticeable downregulation. Zn exposure also impacted the extracellular space associated genes with a similar degree of downregulation. However, cellular compartments such as the lysosome and lytic vacuoles were exclusively enriched with Cu exposure. Moreover, Cu exposure affected various molecular functions such as peroxidase activity, antioxidant activity, and heme binding. Genes associated with both peroxidase and antioxidant activity showed substantial downregulation (Fig. 5B). In contrast, Zn exposure had a more distinct effect, with significant alterations in metallocarboxypeptidase activity and carboxypeptidase activity. All genes associated with metallocarboxypeptidase activity displayed downregulation (Fig. 5C).

**Fig. 6:**
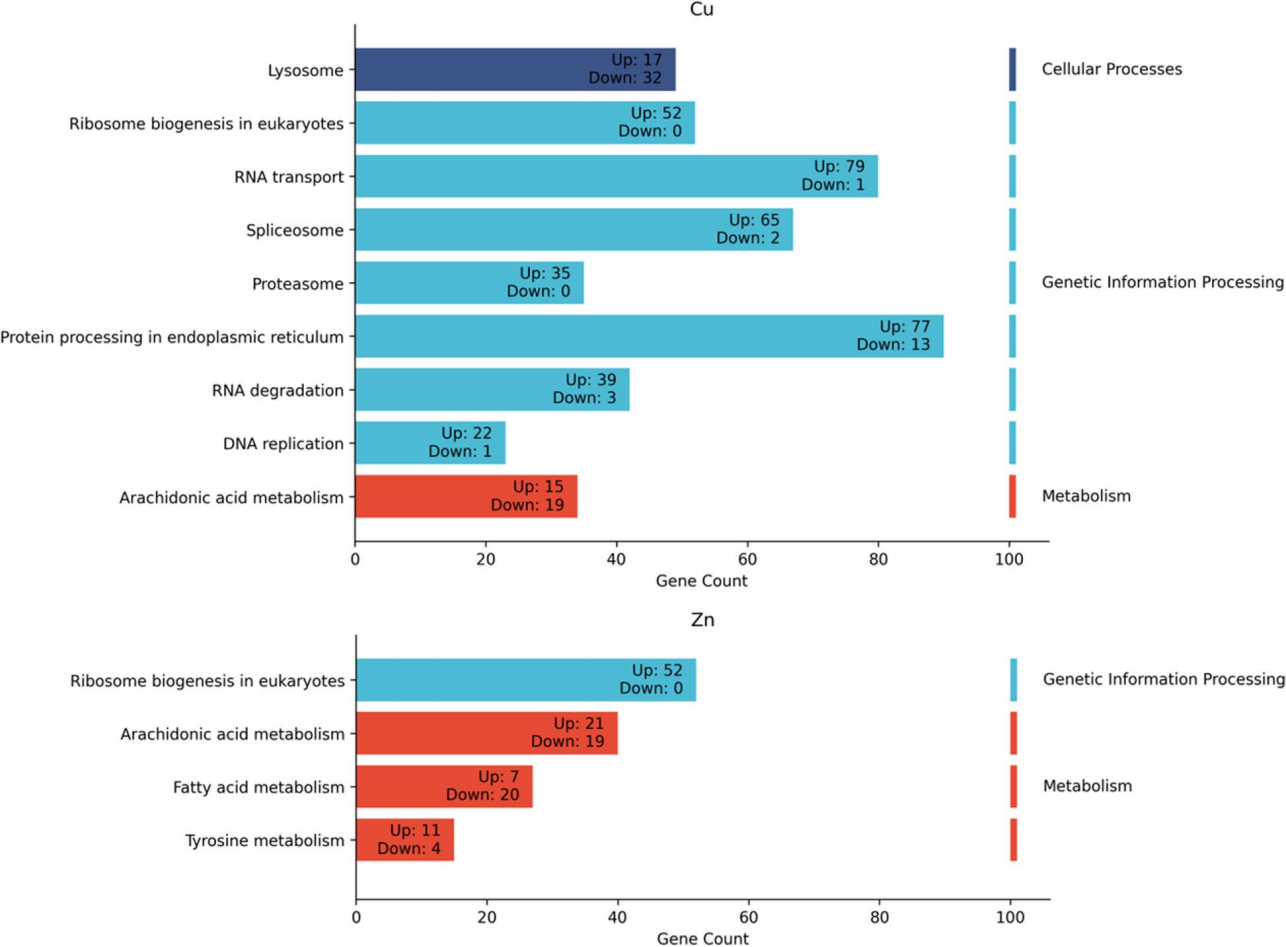
KEGG analysis and identified affected pathways. Enriched pathways are categorized according to their functional relationships using hierarchical terms. Additionally, the number of upregulated or downregulated genes within each pathway is indicated within the corresponding bars

The KEGG pathway analyses revealed differential gene expression profiles elicited by exposure to Cu and Zn across a variety of biological pathways (Fig. 6). Upregulation of genes in response to Cu exposure was observed predominantly within pathways associated with genetic information processing. These encompassed processes, including RNA transport, spliceosome activity, and protein processing within the endoplasmic reticulum. Notably, certain cellular processes, including lysosome function, were also enriched, indicating a broad cellular response to Cu exposure. Conversely, Zn exposure resulted in a significant shift in gene expression within metabolic pathways. This was particularly evident in pathways related to arachidonic acid metabolism and fatty acid metabolism. Moreover, ribosome biogenesis in the eukaryote’s pathway (categorized under genetic information processing) showed significant upregulation of genes in response to both exposures.

Using the STRING database, 94 significantly enriched Reactome pathways were identified as a result of Cu exposure. Similarly, 6 significantly enriched Reactome pathways were identified due to Zn exposure (Table 5). Among the 94 pathways enriched by Cu exposure, 93 were predominated by upregulated genes. These pathways are related to oxidative stress and DNA damage, altered cell cycle progression and DNA replication, and impaired gene expression and RNA metabolism. Oxygen-dependent proline hydroxylation of Hypoxia-inducible Factor Alpha and regulation of ornithine decarboxylase suggest that Cu exposure may induce oxidative stress in *Daphnia magna*. This was also evident by pathways involved in DNA replication and the regulation of the cell cycle checkpoints, which can arrest the cell cycle in response to DNA damage or other stress signals. Cu exposure also enriched the pathways associated with activation and signaling of nuclear factor kappa-B (NF-κB) in B cells and cross-presentation of soluble exogenous antigens (endosomes). These pathways were associated with activation of inflammatory and adaptive immune responses. The interleukin-1 signaling, which is associated with production of ceruloplasmin, a major Cu store was also enriched. On the other hand, genes upregulated with Zn exposure enriched tyrosine metabolism, which involves the biosynthesis and degradation of tyrosine and its derivatives. The major pathway of rRNA processing in the nucleolus and cytosol was the only pathway affected by overexpressed genes following Cu and Zn exposure. Developmental processes and Choline catabolism were overrepresented by down-regulated genes in Cu and Zn exposures, respectively.

## Discussion

*Daphnia magna* is a commonly used model organism for toxicity testing due to its ecological relevance and sensitivity to contaminants such as Cu and Zn [3]. The OECD 202 immobilization assay is used to assess the acute toxicity of contaminants on *Daphnia magna *[27]*.* In this study, an extended 96 h immobilization assay was used to evaluate the effects of Cu and Zn on the mobility of *Daphnia magna*. As both Cu and Zn are essential metals and required for several biological processes, exposure concentrations of less than 100 μg/L did not result in any significant immobilization and was in line with previous reports [28]. Cu concentrations of 133 μg/L and higher resulted in significant immobilization of the test organisms compared to the control groups. The concentration–response curve for Cu showed a clear dose–response relationship, with increasing concentrations of Cu resulting in a proportional increase in immobilization. This was consistent with previous studies that have demonstrated the acute toxicity of Cu on aquatic organisms, including *Daphnia magna* [4, 11, 29, 30]. The calculated IC_5_ value for Cu indicates that Cu contamination at concentrations just above 100 μg/L can have a significant impact on the mobility of aquatic organisms. This suggests that the distinction between the homeostatic boundaries of a healthy environment and a toxic one lies within a very narrow range of Cu concentrations. Therefore, the IC_5_ value is an important metric for establishing regulatory limits for contaminants in aquatic ecosystems and for assessing the risk of toxicity to aquatic organisms with established standards by regulating bodies [31].

The concentration–response curve for Zn indicated a lower sensitivity of *Daphnia magna* to Zn than to Cu. Although Zn is an essential micronutrient for living organisms, excessive accumulation in the environment can cause severe toxicity to aquatic organisms, including the freshwater planktonic crustacean *Daphnia magna* The IC_5_ of Zn was determined to be two times more than the IC_5_ of Cu after 96 h exposure. The results show that Zn contamination has a lower impact on the mobility of aquatic organisms as compared to Cu. These results are in line with previous research using genus *Daphnia* where Zn has been shown to be two to ten times more toxic than Cu [32]. The IC_5_ value used in this study also has practical applications in setting regulatory limits in rivers and other streams found downstream of mining operations where effluents increase the concentrations of these metals. We have previously shown the effects of streams contaminated with effluents containing different metals from decommissioned mining sites on several organisms including *Daphnia magna* [4, 33, 34].

To further understand the underlying mechanisms of this differential toxicity, transcriptomic analysis of *Daphnia magna* has allowed researchers to investigate the molecular responses to Cu and Zn exposure. Several studies have identified differentially expressed genes (DEGs) in *Daphnia magna* exposed to Cu and Zn. This includes genes involved in oxidative stress response, metal ion binding, and detoxification. These genes were upregulated in response to Cu and Zn exposure as observed following quantitative real-time PCR (qPCR) validation of the gene expression [35, 36].

In the present study, *aadc* was identified as the most upregulated gene for both Cu and Zn exposure. It is responsible for the decarboxylation step by converting L-3,4-dihydroxyphenylalanine (L-DOPA) and L-5-hydroxytryptophan (5-HTP) into dopamine and serotonin, respectively in both the catecholamine and the indolamine synthetic pathways, which could result in decreased response to stimuli and decreased mobility [37–40]. Previous studies have shown neurotoxicity effect of nickel, lead, and mercury in humans and rat pheochromocytoma cell line (PC12) through disruption of dopamine synthesis and transport [41, 42]. The relationship between Zn and *aadc* has not been reported in previous studies. The *aadc* expression is upregulated during the innate immune response in *Drosophila melanogaster* [43]. It is also expressed in a wide range of invertebrates, where it plays a critical role in the biosynthesis of biogenic amines, including dopamine, serotonin, and octopamine [37, 40]. These neurotransmitters are involved in regulating physiological processes in invertebrates, including behavior, development, and reproduction. When metal homeostasis is disrupted by excess Cu or Zn, it can cause neurotoxicity in marine invertebrates by inducing oxidative stress, disrupting calcium signaling, and impairing mitochondrial function through the production of reactive oxygen species (ROS) and interaction with sulfhydryl chemical groups [44, 38, 45–47]. The *nrf* is another common gene that was upregulated significantly by both exposures. NRF has been identified in *Caenorhabditis elegans* to produce resistance to nose contractions caused by the widely used antidepressant, fluoxetine [48]. Atli and Sevgiler reported increased oxidative stress in *Daphnia magna* exposed to the antidepressant fluoxetine (20–41 µg/L) and Zn (40–80 µg/L) [49]. Although the regulation of both *aadc* and *nrf* in metal induced toxicity are unclear, the present results suggest that they are regulated by metal exposures in *Daphnia magna*.

Exposure to Zn upregulated the expression of several newly identified biomarker genes such as *clca2*, *enpp4*, *znt1*, and *mt-a*. These genes may play important roles in maintaining homeostasis within cells exposed to excess Zn, either by excreting Zn ions from cells or by reducing influx through membrane transporters [50] *clca2* encodes a calcium-activated chloride channel that allows the transport of chloride and other anions into the cell to maintain the electrochemical balance across the cell membrane [51]. These chloride channels are known to be involved in the secretion of electrolytes and water in epithelial cells, as well as regulating smooth muscle contraction and neuronal excitability in sensory neurons [52, 53]. Bouron and Oberwinkler argued that such channels may also take part in plasmalemmal transport of Zn in *Daphnia magna* [54]. *enpp4* encodes an ectonucleotide pyrophosphatase/phosphodiesterase belonging to the Phospho-/Sulfo-coordinating Metalloenzyme superfamily, which require Zn ions to produce nucleoside-monophosphates from nucleotides and their derivatives [55]. Increased expression of *enpp4* may increase bone mineralization and vascular smooth muscle calcification through hydrolysis of inorganic pyrophosphate, as well as generate extracellular nucleosides for cellular uptake, as seen in mouse [50, 56]. This may lead to immobilization of the animals. In *Xenopus laevis* and rainbow trout, *znt1* encodes a Zn transporter that pumps Zn ions out of the cytoplasm to reduce the Zn concentration in cells [57, 58]. Several studies have reported similar findings, showing that exposure to Zn can upregulate the expression of genes involved in Zn transport and detoxification in marine copepods and fish [57–60]. Taken together, the upregulation of *clca2, enpp4* and *znt1* could indicate an increased cellular activity induced by excess intracellular Zn which will ultimately be sequestrated by metallothionines (MT), mainly MT-A [4, 61].

*Daphnia magna* has a sophisticated detoxification system to deal with the toxicity of heavy metals, which includes the synthesis of MT proteins [62]. MTs are a group of low-molecular-weight, cysteine-rich proteins that bind to heavy metals and play a critical role in the detoxification process [63]. MTs not only bind to metals for the purpose of excretion but also serve as a Zn pool that can be used in the event of deficiency [64]. *Daphnia magna* has three MT isoforms, namely *mt-a*, *mt-b*, and *mt-c* [65]. Although the physiological roles of these MT isoforms during Zn and Cu exposure in *Daphnia magna* have been studied in several experiments, the specific functions of each isoform are still not fully understood [66]. Upregulation of the *mt-a* gene indicates that *Daphnia magna* actively tries to sequester excess Zn. While the expression of *mt-a* was induced by Zn exposure, *mt-b* expression was induced by both Zn and Cu exposure, albeit to a lesser extent than *mt-a* induction by Zn. In contrast, *mt-c* expression was not altered by either exposure. The higher response of *mt-a* to Zn than Cu is consistent with previous research on MTs [61]. Furthermore, *mt-a* appears to be more responsive to Zn exposure than *mt-b* and *mt-c*, as its expression is induced at lower Zn concentrations. In contrast to our results where only *mt-b*, and not *mt-c,* was upregulated by Cu, others have observed that both *mt-b* and *mt-c* expression are induced at higher Cu concentrations [67]. Thus, *mt-a* appears to be the main isoform that sequesters Zn while *mt-b* is induced to a lesser extent by both Cu and Zn. Unlike Cu, the redox-inert nature of Zn supports an evolutionary conservation of Zn binding sites in metalloproteins specializing in sequestering Zn only [68]. It has been suggested that this specificity might be a consequence of necessarily strict control of Zn levels since it is required for a vast number of proteins [69]. The differential expression of *mt-a* and *mt-b* in response to Zn and Cu exposure suggests that they are regulated differently, which might be a result of having different metal transcription factor binding sites in their enhancer regions [70, 71]

The expression levels of *mthfr* and *tsl* genes were found to be upregulated when *Daphnia magna* was exposed to IC_5_ Cu. MTHFR and TSL are important components in DNA synthesis [72]. The *mthfr* encodes for methylenetetrahydrofolate reductase, which plays a crucial role in folate metabolism [73, 74] This enzyme is involved in the conversion of 5,10-methylenetetrahydrofolate to 5-methyltetrahydrofolate [73], a reaction that is necessary for the synthesis of methionine from homocysteine. On the other hand, *tsl* encodes for thymidylate synthase-like protein, which is involved in folate metabolism during DNA synthesis [75]. Thymidylate synthase is an essential enzyme for DNA synthesis as it provides the sole intracellular de novo source of dTMP (deoxythymidine monophosphate), which is subsequently phosphorylated to dTTP for DNA replication [75]. The upregulation of both genes could suggest an increased demand for DNA synthesis or repair in *Daphnia magna* when exposed to IC_5_ Cu. Intracellular accumulation of Cu, Zn, and other heavy metals and their toxicity through increased production of ROS (reactive oxygen species) coupled with reduced antioxidant activity, DNA damage and inhibition of relevant repair mechanisms, and protein misfolding disorders in intertidal copepod, *Daphnia magna* and other eukaryotic organisms are well established [76–79].

The expression levels of metalloendopeptidase-like protein (*mep)* and mucin (*muc)* genes were downregulated in *Daphnia magna* following exposure to IC_5_ Cu. These genes are involved in the degradation of extracellular matrix proteins, which are a major component of the mucus layer in the gastrointestinal tract [80–82]. The observed downregulation of *mep* and *muc* genes indicates a potential decrease in both the degradation and production of mucus within the gastrointestinal tract of *Daphnia magna* [83]. This could potentially lead to changes in the organism’s absorption and digestion of nutrients, as well as the ability to protect itself from harmful substances in the environment [84–86].

Biogenic amines are low-molecular-weight, organic nitrogen compounds formed either by the decarboxylation of amino acids or by the amination and transamination of aldehydes and ketones during normal metabolic processes in living cells [87]. They participate in signaling as neurotransmitters and hormones, or as components of vitamins, phospholipids, and ribosomes within cells. Biogenic amines also play a number of important roles in physiological processes, from cell proliferation and differentiation to nutrition, immune response, and neurobiology and reproduction [88]. AADCs catalyze the conversion of aromatic L-amino acids into aromatic monoamines [89]. S-adenosylmethionine decarboxylase (AMD) is an essential regulatory component of the polyamine biosynthetic pathway by generating the n-propylamine residue required for the synthesis of spermidine and spermine from putrescin [90]. The upregulation of these central genes indicates a potential need for biogenic amines in the presence of these metals, possibly due to their essential roles in cellular development, metabolism, and ribosomal function.

Peroxidases are enzymes that typically catalyze a reaction where a peroxide (ROOR’) is reduced to water (ROH) or alcohol (R’OH) using an electron donor. This reaction is crucial in lignification, suberization, auxin catabolism, and the response to environmental stresses like wounding, pathogen attack, and oxidative stress [91]. In this study, many genes related to peroxidase activity were downregulated in the presence of both Cu and Zn, possibly as a response to oxidative stress. Cu exposure has been reported to induce changes in the expression of glutathione-related genes, which play a crucial role in protecting cells from oxidative stress [92]. Peroxidases participate in the detoxification of reactive oxygen species (ROS), and their activity is modulated in response to changes in ROS levels.

Cu exposure resulted in the regulation of detoxification processes and several molecular functions, while Zn exposure demonstrated a mix of up- and downregulation in several processes and functions. Cu is a component of enzymes involved in energy production, iron metabolism, neurotransmitter biosynthesis, and connective tissue formation [93]. Cu is also needed for lysosomal, waste removal and recycling within the cell [93]. This role of Cu could explain enrichment in terms related to lysosomes and lytic vacuoles in the presence of Cu. Moreover, signaling and activation of the NF-κB, and cross-presentation of soluble exogenous antigens (endosomes), could be associated with the role of Cu in enhancing immune activity of macrophages, a phenomenon known as immune priming [94, 95]. Cu is also involved in cellular processes both at the cell surface and in cell signaling, which could explain the enrichment of terms related to the plasma membrane [96]. The apical part of a cell is a specialized region involved in secretion and absorption that may be influenced by the presence of Cu. Zn is essential for enzyme function, protein structure, and gene regulation. However, its roles may differ from those of Cu, leading to the enrichment of different cellular components as seen in the functional enrichment results.

KEGG pathway enrichment resulted in distinct pathways for each exposure. Cell toxicity may occur due to high concentrations of Cu, either from environmental intake or abnormal accumulation within cells as a result of genetic mutations [97]. Our results show significant upregulation in genetic information processing pathways, including cell cycle checkpoints, DNA replication, RNA transport, and protein processing in the endoplasmic reticulum, which could be indicative of the cellular response to mitigate Cu induced stress [77, 78]. However, the absence of pathways known to increase the expression of antioxidant and anti-inflammatory enzymes indicates that the oxidative stress may be uncontrolled and potentially lead to cellular damage. This is also supported by enrichment of the interleukin-1 signaling, which induces the major Cu sequestrating protein, ceruloplasmin [98]. On the other hand, Zn is known to influence gene expression through both direct and indirect interactions. Direct interactions involve the binding of Zn to transcription factors, thereby altering the rate of transcription, while indirect interactions may involve signal transduction systems and hormonal or cytokine stimuli [99]. We observed an upregulation in genes associated with metabolic pathways following Zn exposure which indicates the activation of certain regulatory mechanisms. Evidence for this regulation is provided by the enrichment of the tyrosine metabolism pathway. Tyrosine metabolism pathway produces various bioactive molecules from Tyrosine, which serve multiple functions. They regulate antioxidant defense, act as chelators of metal ions (including Zn) and modulate the availability and toxicity of these ions [100, 101]. Moreover, Zn has been shown to affect the activity and expression of antioxidant enzymes, further emphasizing the role of Zn in detoxification processes [102]. Eukaryotic cells have been shown to rapidly modulate ribosome biogenesis in response to oxidative stress, DNA damage, and alterations in amino acid levels [103]. The upregulation of genes within the ‘ribosome biogenesis in eukaryotes’ pathway, observed following both exposures, indicates a stress response. Enrichment of ribosome-related pathways has been reported after exposure to metal stress, carbamates, oxidative stress and *microcystis* exposure in *Daphnia pulex* and *Daphnia magna* [104–106]. Under stress conditions the rRNA processing is inhibited and the unprocessed rRNA is stored in the nucleolus until the stress is resolved. This allows the cells to conserve energy and maintain the integrity of the nucleolus, which is a key organelle for ribosome biogenesis and stress response [107]. Activation of the stimuli-sensing channels may activate the Ca^2+^ dependent endonuclease, which can cleave the rRNA and induce nucleolar fragmentation [108]. This may result in reduced ribosome biogenesis and protein synthesis as well as increased apoptosis.

The transcriptomics analysis of *Daphnia magna* exposed to IC_5_ concentrations of Cu and Zn revealed significant pathway enrichment that has direct implications for both individual organisms and population dynamics. Collectively, these molecular disturbances can decrease population resilience, reduce reproductive success, and alter population dynamics, ultimately impacting ecosystem stability [77, 104]. Demonstrating significant pathway enrichment at the IC_5_ level is crucial as it highlights the initial molecular disruptions that serve as early indicators of ensuing physiological and phenotypic changes, providing a predictive model for more severe impacts at higher concentrations, and offering valuable insights for regulatory frameworks to preemptively mitigate environmental and ecological risks.

Overall, transcriptomics analysis provided valuable insights into the molecular mechanisms underlying the responses of *Daphnia magna* to Cu and Zn exposure. In the present study we identify key genes and pathways involved in stress responses, providing a basis for further investigations into the effects of heavy metal exposure on aquatic organisms. In addition, we have identified IC_5_ values for *Daphnia magna,* which increases the resolution of our understanding of the organism’s response to environmental stressors.

In conclusion, our findings provide valuable insights into the distinct cellular responses invoked by Cu and Zn exposure. This approach not only expands our comprehension of the impact of these metals on *Daphnia magna*, but also establishes a robust foundation for prospective investigations into this key aquatic organism.

### Supplementary Information

Supplementary Material 1

## Data Availability

The dataset used and analyzed in the current study is available from the ENA database under study accession PRJNA1084256.

## References

[1] Ebert D. Daphnia as a versatile model system in ecology and evolution. EvoDevo. 2022;13(1):16. 35941607 10.1186/s13227-022-00199-0 PMC9360664

[2] Jansen M, Coors A, Stoks R, De Meester L. Evolutionary ecotoxicology of pesticide resistance: a case study in Daphnia. Ecotoxicology. 2011;20(3):543–51. 21380529 10.1007/s10646-011-0627-z

[3] Kim HJ, Koedrith P, Seo YR. Ecotoxicogenomic approaches for understanding molecular mechanisms of environmental chemical toxicity using aquatic invertebrate, daphnia model organism. Int J Mol Sci. 2015;16:12261–87. 10.3390/ijms160612261. 26035755 10.3390/ijms160612261 PMC4490443

[4] Paylar B, Bezabhe YH, Mangu JCK, Thamke V, Igwaran A, Modig C, et al. Assessing organism differences in mixed metal sensitivity. Sci Total Environ. 2023;905: 167340. 37751843 10.1016/j.scitotenv.2023.167340

[5] Bonham M, O’Connor JM, Hannigan BM, Strain JJ. The immune system as a physiological indicator of marginal copper status? Br J Nutr. 2002;87(5):393–403. 12010579 10.1079/BJNBJN2002558

[6] Tsang T, Davis CI, Brady DC. Copper biology. Curr Biol. 2021;31(9):R421–7. 33974864 10.1016/j.cub.2021.03.054

[7] Sangeetha VJ, Dutta S, Moses JA, Anandharamakrishnan C. Zinc nutrition and human health: overview and implications. eFood. 2022;3(5):e17.

[8] Hodgkinson V, Petris MJ. Copper homeostasis at the host-pathogen interface. J Biol Chem. 2012;287(17):13549–55. 22389498 10.1074/jbc.R111.316406 PMC3340201

[9] Maret W. Zinc biochemistry: from a single zinc enzyme to a key element of life. Adv Nutr. 2013;4(1):82–91. 23319127 10.3945/an.112.003038 PMC3648744

[10] Achard-Joris M, Moreau J-L, Lucas M, Baudrimont M, Mesmer-Dudons N, Gonzalez P, et al. Role of metallothioneins in superoxide radical generation during copper redox cycling: Defining the fundamental function of metallothioneins. Biochimie. 2007;89(12):1474–88. 17681660 10.1016/j.biochi.2007.06.005

[11] Paylar B, Asnake S, Sjöberg V, Ragnvaldsson D, Jass J, Olsson P-E. Influence of water hardness on zinc toxicity in Daphnia magna. J Appl Toxicol. 2022;42(9):1510–23. 35285959 10.1002/jat.4319 PMC9543215

[12] Jankowski MD, Fairbairn DJ, Baller JA, Westerhoff BM, Schoenfuss HL. Using the Daphnia magna Transcriptome to Distinguish Water Source: Wetland and Stormwater Case Studies. Environ Toxicol Chem. 2022;41(9):2107–23. 35622010 10.1002/etc.5392 PMC9545677

[13] Andrzejczyk N. Transcriptomics in Ecotoxicology: Characterizing the Effects of Contaminants and Environmental Parameters on Aquatic Populations. Riverside: University of California; 2020.

[14] Lowe R, Shirley N, Bleackley M, Dolan S, Shafee T. Transcriptomics technologies. PLoS Comput Biol. 2017;13(5): e1005457. 28545146 10.1371/journal.pcbi.1005457 PMC5436640

[15] Roh JY, Sim SJ, Yi J, Park K, Chung KH, Ryu DY, et al. Ecotoxicity of silver nanoparticles on the soil nematode Caenorhabditis elegans using functional ecotoxicogenomics. Environ Sci Technol. 2009;43(10):3933–40. 19544910 10.1021/es803477u

[16] Supplitt S, Karpinski P, Sasiadek M, and Laczmanska I. Current Achievements and Applications of Transcriptomics in Personalized Cancer Medicine. Int J Mol Sci. 2021;22(3):1422 10.3390/ijms22031422 PMC7866970 33572595

[17] Poynton HC, Loguinov AV, Varshavsky JR, Chan S, Perkins EJ, Vulpe CD. Gene expression profiling in Daphnia magna part I: concentration-dependent profiles provide support for the No Observed Transcriptional Effect Level. Environ Sci Technol. 2008;42(16):6250–6. 18767695 10.1021/es8010783

[18] Kishor K, Sahu RK. Biomarkers and their applications in toxicology. Lab Anim. 2016;45(3):103–103.

[19] Hagger JA, Jones MB, Lowe D, Leonard DR, Owen R, Galloway TS. Application of biomarkers for improving risk assessments of chemicals under the Water Framework Directive: a case study. Mar Pollut Bull. 2008;56(6):1111–8. 18474377 10.1016/j.marpolbul.2008.03.040

[20] van IDGP, Szuhai K, Briaire-de Bruijn IH, Kostine M, Kuijjer ML, Bovée J. Machine learning analysis of gene expression data reveals novel diagnostic and prognostic biomarkers and identifies therapeutic targets for soft tissue sarcomas. PLoS Comput Biol. 2019;15(2):e1006826. 30785874 10.1371/journal.pcbi.1006826 PMC6398862

[21] Paylar B, Längkvist M, Jass J, Olsson PE. Utilization of Computer Classification Methods for Exposure Prediction and Gene Selection in Daphnia magna Toxicogenomics. Biology (Basel). 2023;12(5):692. 10.3390/biology12050692 PMC10215664 37237504

[22] Jiang Y, Chen J, Wu Y, Wang Q, Li H. Sublethal Toxicity Endpoints of Heavy Metals to the Nematode Caenorhabditis elegans. PLoS ONE. 2016;11(1): e0148014. 26824831 10.1371/journal.pone.0148014 PMC4732754

[23] Anders S, Huber W. Differential expression analysis for sequence count data. Genome Biol. 2010;11(10):R106. 20979621 10.1186/gb-2010-11-10-r106 PMC3218662

[24] Schmittgen TD, Livak KJ. Analyzing real-time PCR data by the comparative C-T method. Nat Protoc. 2008;3(6):1101–8. 18546601 10.1038/nprot.2008.73

[25] Altschul SF, Gish W, Miller W, Myers EW, Lipman DJ. Basic local alignment search tool. J Mol Biol. 1990;215(3):403–10. 2231712 10.1016/S0022-2836(05)80360-2

[26] Szklarczyk D, Kirsch R, Koutrouli M, Nastou K, Mehryary F, Hachilif R, et al. The STRING database in 2023: protein-protein association networks and functional enrichment analyses for any sequenced genome of interest. Nucleic Acids Res. 2023;51(D1):D638-d646. 36370105 10.1093/nar/gkac1000 PMC9825434

[27] OECD, Test No. 202: Daphnia sp. Acute Immobilisation Test, OECD Guidelines for the testing of chemicals, section 2. Paris: OECD Publishing; 2004. 10.1787/9789264069947-en.

[28] Rainbow P. Heavy metal levels in marine invertebrates. In: Furness RW, Rainbow PS, editors. Heavy metals in the marine environment. CRC Press; 2018: p. 67–79.

[29] Chupani L, Sjöberg V, Jass J, Olsson PE. Water Hardness Alters the Gene Expression Response and Copper Toxicity in Daphnia magna. Fishes. 2022;7(5):248.

[30] Cooper NL, Bidwell JR, Kumar A. Toxicity of copper, lead, and zinc mixtures to Ceriodaphnia dubia and Daphnia carinata. Ecotoxicol Environ Saf. 2009;72(5):1523–8. 19419764 10.1016/j.ecoenv.2009.03.002

[31] OECD, Guidance Document on Aquatic Toxicity Testing of Difficult Substances and Mixtures OECD Series on Testing and Assessment, OECD Publishing, Paris, 10.1787/0ed2f88e-en. Testing and Assessment. 2019.

[32] Pereira CMS, Deruytter D, Blust R, De Schamphelaere KAC. Effect of temperature on chronic toxicity of copper, zinc, and nickel to Daphnia magna. Environ Toxicol Chem. 2017;36(7):1909–16. 27976806 10.1002/etc.3714

[33] Kumar R, Pradhan A, Khan FA, Lindström P, Ragnvaldsson D, Ivarsson P, et al. Comparative Analysis of Stress Induced Gene Expression in Caenorhabditis elegans following Exposure to Environmental and Lab Reconstituted Complex Metal Mixture. PLoS ONE. 2015;10(7): e0132896. 26168046 10.1371/journal.pone.0132896 PMC4500601

[34] Pradhan A, Ivarsson P, Ragnvaldsson D, Berg H, Jass J, Olsson PE. Transcriptional responses of zebrafish to complex metal mixtures in laboratory studies overestimates the responses observed with environmental water. Sci Total Environ. 2017;584–585:1138–46. 28159303 10.1016/j.scitotenv.2017.01.174

[35] Poynton HC, Taylor NS, Hicks J, Colson K, Chan S, Clark C, et al. Metabolomics of microliter hemolymph samples enables an improved understanding of the combined metabolic and transcriptional responses of Daphnia magna to cadmium. Environ Sci Technol. 2011;45(8):3710–7. 21417318 10.1021/es1037222

[36] Qi Q, Li Q, Li J, Mo J, Tian Y, Guo J. Transcriptomic analysis and transgenerational effects of ZnO nanoparticles on Daphnia magna: Endocrine-disrupting potential and energy metabolism. Chemosphere. 2022;290: 133362. 34933032 10.1016/j.chemosphere.2021.133362

[37] Zhu MY, Juorio AV. Aromatic l-amino acid decarboxylase: Biological characterization and functional role. General Pharmacology: The Vascular System. 1995;26(4):681–96. 7635243 10.1016/0306-3623(94)00223-a

[38] Lutsenko S, Washington-Hughes C, Ralle M, Schmidt K. Copper and the brain noradrenergic system. J Biol Inorg Chem. 2019;24(8):1179–88. 31691104 10.1007/s00775-019-01737-3 PMC6941745

[39] Nasrin S, Ichinose H, Nagatsu T. Comparison of characteristics of bovine aromatic L-amino acid decarboxylase with human enzyme. Biochim Biophys Acta. 1992;1118(3):318–22. 1737055 10.1016/0167-4838(92)90291-k

[40] Bellot, M., M. Faria, C. Gómez-Canela, D. Raldúa, and C. Barata Pharmacological Modulation of Behaviour, Serotonin and Dopamine Levels in Daphnia magna Exposed to the Monoamine Oxidase Inhibitor Deprenyl. Toxics, 2021. 9, 10.3390/toxics9080187. 10.3390/toxics9080187 PMC8402476 34437505

[41] Hui C. Effects of mercury on the dopamine transporter cell surface expression in PC12 cells. CUNY Academic Works; 2019. p. 918. https://academicworks.cuny.edu/jj_etds/131.

[42] Wezynfeld, N.E., A.M. Bonna, D. Płonka, W. Bal, and T. Frączyk Ni(II) Ions May Target the Entire Melatonin Biosynthesis Pathway—A Plausible Mechanism of Nickel Toxicity. Molecules, 2022. 27, 10.3390/molecules27175582. 10.3390/molecules27175582 PMC9458082 36080347

[43] Davis MM, Primrose DA, Hodgetts RB. A member of the p38 mitogen-activated protein kinase family is responsible for transcriptional induction of Dopa decarboxylase in the epidermis of Drosophila melanogaster during the innate immune response. Mol Cell Biol. 2008;28(15):4883–95. 18519585 10.1128/MCB.02074-07 PMC2493365

[44] Deidda, I., R. Russo, R. Bonaventura, C. Costa, F. Zito, and N. Lampiasi Neurotoxicity in Marine Invertebrates: An Update. Biology, 2021. 10, 10.3390/biology10020161. 10.3390/biology10020161 PMC7922589 33670451

[45] Chasapis CT, Loutsidou AC, Spiliopoulou CA, Stefanidou ME. Zinc and human health: an update. Arch Toxicol. 2012;86(4):521–34. 22071549 10.1007/s00204-011-0775-1

[46] Morris DR, Levenson CW. Neurotoxicity of Zinc. In: Aschner M, Costa LG, editors. Neurotoxicity of Metals. Cham: Springer International Publishing; 2017. p. 303–12.

[47] Carmona A, Roudeau S, Ortega R. Molecular Mechanisms of Environmental Metal Neurotoxicity: A Focus on the Interactions of Metals with Synapse Structure and Function. Toxics. 2021;9(9):198. 10.3390/toxics9090198 PMC8471991 34564349

[48] Choy RK, Kemner JM, Thomas JH. Fluoxetine-resistance genes in Caenorhabditis elegans function in the intestine and may act in drug transport. Genetics. 2006;172(2):885–92. 16118202 10.1534/genetics.103.024869 PMC1456238

[49] Atli G, Sevgiler Y. Binary effects of fluoxetine and zinc on the biomarker responses of the non-target model organism Daphnia magna. Environ Sci Pollut Res. 2024;31(19):27988–8006. 10.1007/s11356-024-32846-5 PMC11058962 38528217

[50] Villa-Bellosta R, Wang X, Millán JL, Dubyak GR, O’Neill WC. Extracellular pyrophosphate metabolism and calcification in vascular smooth muscle. Am J Physiol Heart Circ Physiol. 2011;301(1):H61–8. 21490328 10.1152/ajpheart.01020.2010 PMC3129914

[51] Barish ME. A transient calcium-dependent chloride current in the immature Xenopus oocyte. J Physiol. 1983;342:309–25. 6313909 10.1113/jphysiol.1983.sp014852 PMC1193960

[52] Huang F, Wong X, Jan LY. International union of basic and clinical pharmacology. LXXXV: calcium-activated chloride channel. Pharmacol Rev. 2012;64(1):1–15. 22090471 10.1124/pr.111.005009 PMC3250081

[53] Hartzell C, Putzier I, Arreola J. Calcium-activated chloride channels. Annu Rev Physiol. 2005;67:719–58. 15709976 10.1146/annurev.physiol.67.032003.154341

[54] Bouron A, Oberwinkler J. Contribution of calcium-conducting channels to the transport of zinc ions. Pflügers Arch Eur J Physiol. 2014;466(3):381–7. 23719866 10.1007/s00424-013-1295-z

[55] Gijsbers R, Ceulemans H, Stalmans W, Bollen M. Structural and Catalytic Similarities between Nucleotide Pyrophosphatases/Phosphodiesterases and Alkaline Phosphatases*. J Biol Chem. 2001;276(2):1361–8. 11027689 10.1074/jbc.M007552200

[56] Johnson K, Polewski M, van Etten D, Terkeltaub R. Chondrogenesis mediated by PPi depletion promotes spontaneous aortic calcification in NPP1-/- mice. Arterioscler Thromb Vasc Biol. 2005;25(4):686–91. 15625282 10.1161/01.ATV.0000154774.71187.f0

[57] Rivero M, Marín-Barba M, Gutiérrez L, Lozano-Velasco E, Wheeler GN, Sánchez-Marcos J, et al. Toxicity and biodegradation of zinc ferrite nanoparticles in Xenopus laevis. J Nanopart Res. 2019;21(8):181.

[58] Walker PA, Kille P, Hurley A, Bury NR, Hogstrand C. An in vitro method to assess toxicity of waterborne metals to fish. Toxicol Appl Pharmacol. 2008;230(1):67–77. 18394669 10.1016/j.taap.2008.02.012

[59] Kim RO, Choi JS, Kim BC, Kim WK. Comparative Analysis of Transcriptional Profile Changes in Larval Zebrafish Exposed to Zinc Oxide Nanoparticles and Zinc Sulfate. Bull Environ Contam Toxicol. 2017;98(2):183–9. 27995293 10.1007/s00128-016-1995-0

[60] Banae M, Zeidi A ,Mikušková N, Faggio C. Assessing Metal Toxicity on Crustaceans in Aquatic Ecosystems: A Comprehensive Review. Biological Trace Element Research. 2024. 10.1007/s12011-024-04122-7. 10.1007/s12011-024-04122-7 38472509

[61] Arao T, Kato Y, Nong QD, Yamamoto H, Watanabe H, Matsuura T, et al. Production of genome-edited Daphnia for heavy metal detection by fluorescence. Sci Rep. 2020;10(1):21490. 33293611 10.1038/s41598-020-78572-z PMC7722880

[62] Amiard JC, Amiard-Triquet C, Barka S, Pellerin J, Rainbow PS. Metallothioneins in aquatic invertebrates: Their role in metal detoxification and their use as biomarkers. Aquat Toxicol. 2006;76(2):160–202. 16289342 10.1016/j.aquatox.2005.08.015

[63] George SG, Olsson P-E. Metallothioneins as indicators of trace metal pollution. In: Kramer KJM, editor. Biomonitoring of coastal waters and estuaries. Boca Raton: CRC Press; 1994. p. 151–78.

[64] Hoadley JE, Leinart AS, Cousins RJ. Relationship of 65Zn absorption kinetics to intestinal metallothionein in rats: effects of zinc depletion and fasting. J Nutr. 1988;118(4):497–502. 3357065 10.1093/jn/118.4.497

[65] Asselman J, Shaw JR, Glaholt SP, Colbourne JK, De Schamphelaere KAC. Transcription patterns of genes encoding four metallothionein homologs in Daphnia pulex exposed to copper and cadmium are time- and homolog-dependent. Aquatic toxicology (Amsterdam, Netherlands). 2013;142–143:422–30. 24113165 10.1016/j.aquatox.2013.09.010 PMC3891374

[66] Wang X, Liu J, Tan Q, Ren J, Liang D, Fan W. Development of multi-metal interaction model for Daphnia magna: Significance of metallothionein in cellular redistribution. Ecotoxicol Environ Saf. 2018;151:42–8. 29306069 10.1016/j.ecoenv.2017.12.040

[67] Vašák M, Meloni G. Mammalian Metallothionein-3: New Functional and Structural Insights. Int J Mol Sci. 2017;18(6):1117. 10.3390/ijms18061117 PMC5485941 28538697

[68] Maret W. The function of zinc metallothionein: a link between cellular zinc and redox state. J Nutr. 2000;130(5S Suppl):1455s-s1458. 10801959 10.1093/jn/130.5.1455S

[69] Subramanian Vignesh K, Deepe GS Jr. Metallothioneins: Emerging Modulators in Immunity and Infection. Int J Mol Sci. 2017;18(10):2197. 10.3390/ijms18102197 PMC5666878 29065550

[70] Selvaraj A, Balamurugan K, Yepiskoposyan H, Zhou H, Egli D, Georgiev O, et al. Metal-responsive transcription factor (MTF-1) handles both extremes, copper load and copper starvation, by activating different genes. Genes Dev. 2005;19(8):891–6. 15833915 10.1101/gad.1301805 PMC1080128

[71] Rutherford JC, Bird AJ. Metal-responsive transcription factors that regulate iron, zinc, and copper homeostasis in eukaryotic cells. Eukaryot Cell. 2004;3(1):1–13. 14871932 10.1128/EC.3.1.1-13.2004 PMC329510

[72] Etienne MC, Ilc K, Formento JL, Laurent-Puig P, Formento P, Cheradame S, et al. Thymidylate synthase and methylenetetrahydrofolate reductase gene polymorphisms: relationships with 5-fluorouracil sensitivity. Br J Cancer. 2004;90(2):526–34. 14735204 10.1038/sj.bjc.6601523 PMC2409555

[73] Raghubeer S, Matsha TE. Methylenetetrahydrofolate (MTHFR), the One-Carbon Cycle, and Cardiovascular Risks. Nutrients. 2021;13(12):4562. 10.3390/nu13124562 PMC8703276 34960114

[74] Lindeman LC, Thaulow J, Song Y, Kamstra JH, Xie L, Asselman J, et al. Epigenetic, transcriptional and phenotypic responses in two generations of Daphnia magna exposed to the DNA methylation inhibitor 5-azacytidine. Environ Epigenet. 2019;5(3):dvz016. 31528364 10.1093/eep/dvz016 PMC6736351

[75] Myllykallio H, Lipowski G, Leduc D, Filee J, Forterre P, Liebl U. An Alternative Flavin-Dependent Mechanism for Thymidylate Synthesis. Science. 2002;297(5578):105–7. 12029065 10.1126/science.1072113

[76] Kim B-M, Rhee J-S, Jeong C-B, Seo JS, Park GS, Lee Y-M, et al. Heavy metals induce oxidative stress and trigger oxidative stress-mediated heat shock protein (hsp) modulation in the intertidal copepod Tigriopus japonicus. Comp Biochem Physiol C: Toxicol Pharmacol. 2014;166:65–74. 25058597 10.1016/j.cbpc.2014.07.005

[77] Atienzar FA, Cheung VV, Jha AN, Depledge MH. Fitness parameters and DNA effects are sensitive indicators of copper-induced toxicity in Daphnia magna. Toxicol Sci. 2001;59(2):241–50. 11158717 10.1093/toxsci/59.2.241

[78] Martins SG, Zilhão R, Thorsteinsdóttir S, Carlos AR. Linking Oxidative Stress and DNA Damage to Changes in the Expression of Extracellular Matrix Components. Front Genet. 2021;12: 673002. 34394183 10.3389/fgene.2021.673002 PMC8358603

[79] Sahlmann A, Lode T, Heuschele J, Borgå K, Titelman J, Hylland K. Genotoxic Response and Mortality in 3 Marine Copepods Exposed to Waterborne Copper. Environ Toxicol Chem. 2019;38(10):2224–32. 31343775 10.1002/etc.4541

[80] Rousseau K, Byrne C, Kim YS, Gum JR, Swallow DM, Toribara NW. The complete genomic organization of the human MUC6 and MUC2 mucin genes. Genomics. 2004;83(5):936–9. 15081123 10.1016/j.ygeno.2003.11.003

[81] Brum AM, van der Leije CS, Schreuders-Koedam M, Chaibi S, van Leeuwen JP, van der Eerden BC. Mucin 1 (Muc1) Deficiency in Female Mice Leads to Temporal Skeletal Changes During Aging. JBMR Plus. 2018;2(6):341–50. 30460337 10.1002/jbm4.10061 PMC6237209

[82] Vasamsetti BMK, Chon K, Choi J-Y, Kim J, Yoon C-Y. Transcriptome Analysis of Thiram-Treated Zebrafish (Danio rerio) Embryos Reveals Disruption of Reproduction Signaling Pathways. Biology. 2023;12(2):156. 36829436 10.3390/biology12020156 PMC9953208

[83] Asselman J, Semmouri I, Jackson CE, Keith N, Van Nieuwerburgh F, Deforce D, et al. Genome-Wide Stress Responses to Copper and Arsenic in a Field Population of Daphnia. Environ Sci Technol. 2019;53(7):3850–9. 30817885 10.1021/acs.est.8b06720

[84] Hearn J, Clark J, Wilson PJ, Little TJ. Daphnia magna modifies its gene expression extensively in response to caloric restriction revealing a novel effect on haemoglobin isoform preference. Mol Ecol. 2020:29(17):3261–76. 10.1111/mec.15557 32687619

[85] Schwarzenberger A, Courts C, von Elert E. Target gene approaches: Gene expression in Daphnia magna exposed to predator-borne kairomones or to microcystin-producing and microcystin-free Microcystis aeruginosa. BMC Genomics. 2009;10(1):527. 19917101 10.1186/1471-2164-10-527 PMC2784803

[86] Qiu TA, Bozich JS, Lohse SE, Vartanian AM, Jacob LM, Meyer BM, et al. Gene expression as an indicator of the molecular response and toxicity in the bacterium Shewanella oneidensis and the water flea Daphnia magna exposed to functionalized gold nanoparticles. Environ Sci Nano. 2015;2(6):615–29.

[87] Erdag D, Merhan O, Yildiz B. Biochemical and Pharmacological Properties of Biogenic Amines, In Proestos C, editor. Biogenic Amines. IntechOpen. 2019. 10.5772/intechopen.81569.

[88] Rodríguez-López R, Morales M, Sánchez-Jiménez F. Histamine and Its Receptors as a Module of the Biogenic Amine Diseasome. In: Blandina P, Passani MB, editors. Histamine Receptors: Preclinical and Clinical Aspects. Cham: Springer International Publishing; 2016. p. 173–214.

[89] Han S-W, Shin J-S. Aromatic L-amino acid decarboxylases: mechanistic features and microbial applications. Appl Microbiol Biotechnol. 2022;106(12):4445–58. 35763068 10.1007/s00253-022-12028-4

[90] Pegg AE, Xiong H, Feith DJ, Shantz LM. S-Adenosylmethionine decarboxylase: structure, function and regulation by polyamines. Biochem Soc Trans. 1998;26(4):580–6. 10047786 10.1042/bst0260580

[91] Maehly A, Chance B. Catalases and peroxidases. Methods Biochem Anal. 1954;1:357–424. 13193536 10.1002/9780470110171.ch14

[92] Xia J-L, Wu S, Zhang R-Y, Zhang C-G, He H, Jiang H-C, et al. Effects of Copper Exposure on Expression of Glutathione-Related Genes in Acidithiobacillus ferrooxidans. Curr Microbiol. 2011;62(5):1460–6. 21305293 10.1007/s00284-011-9881-9

[93] Madina MH, Rahman MS, Zheng H, Germain H. Vacuolar membrane structures and their roles in plant–pathogen interactions. Plant Mol Biol. 2019;101(4):343–54. 31621005 10.1007/s11103-019-00921-y

[94] Deigendesch N, Zychlinsky A, Meissner F. Copper Regulates the Canonical NLRP3 Inflammasome. J Immunol. 2018;200(5):1607–17. 29358279 10.4049/jimmunol.1700712

[95] Djoko KY, Ong CL, Walker MJ, McEwan AG. The Role of Copper and Zinc Toxicity in Innate Immune Defense against Bacterial Pathogens. J Biol Chem. 2015;290(31):18954–61. 26055706 10.1074/jbc.R115.647099 PMC4521016

[96] Tancini B, Buratta S, Delo F, Sagini K, Chiaradia E, Pellegrino RM, et al. Lysosomal Exocytosis: The Extracellular Role of an Intracellular Organelle. Membranes. 2020;10(12):406. 33316913 10.3390/membranes10120406 PMC7764620

[97] Chen C-H, Chou Y-T, Yang Y-W, Lo K-Y. High-dose copper activates p53-independent apoptosis through the induction of nucleolar stress in human cell lines. Apoptosis. 2021;26(11):612–27. 34708319 10.1007/s10495-021-01692-y

[98] Barber EF, Cousins RJ. Interleukin-1–stimulated induction of ceruloplasmin synthesis in normal and copper-deficient rats. J Nutr. 1988;118(3):375–81. 3258371 10.1093/jn/118.3.375

[99] Cousins RJ. A role of zinc in the regulation of gene expression. Proceedings of the Nutrition Society. 1998;57(2):307–11. 9656334 10.1079/pns19980045

[100] Sreenivasulu K, Raghu P, Nair KM. Polyphenol-rich beverages enhance zinc uptake and metallothionein expression in Caco-2 cells. J Food Sci. 2010;75(4):H123–8. 20546406 10.1111/j.1750-3841.2010.01582.x

[101] Kim EY, Pai TK, Han O. Effect of bioactive dietary polyphenols on zinc transport across the intestinal Caco-2 cell monolayers. J Agric Food Chem. 2011;59(8):3606–12. 21410257 10.1021/jf104260j PMC3087602

[102] Kaznina NM, Batova YV, Repkina NS, Titov AF. Effect of Zinc Deficiency on Gene Expression and Antioxidant Enzyme Activity in Barley Plants at Optimal and Low Temperatures. Biology Bulletin. 2022;49(6):636–44.

[103] Temaj G, Chichiarelli S, Eufemi M, Altieri F, Hadziselimovic R, Farooqi AA, et al. Ribosome-Directed Therapies in Cancer. Biomedicines. 2022;10(9):2088. 10.3390/biomedicines10092088 PMC9495564 36140189

[104] Asselman J, De Coninck DI, Glaholt S, Colbourne JK, Janssen CR, Shaw JR, et al. Identification of pathways, gene networks, and paralogous gene families in Daphnia pulex responding to exposure to the toxic cyanobacterium Microcystis aeruginosa. Environ Sci Technol. 2012;46(15):8448–57. 22799445 10.1021/es301100j PMC3730285

[105] Pereira JL, Hill CJ, Sibly RM, Bolshakov VN, Gonçalves F, Heckmann L-H, et al. Gene transcription in Daphnia magna: Effects of acute exposure to a carbamate insecticide and an acetanilide herbicide. Aquat Toxicol. 2010;97(3):268–76. 20092900 10.1016/j.aquatox.2009.12.023

[106] Vandegehuchte MB, Vandenbrouck T, De Coninck D, De Coen WM, Janssen CR. Gene transcription and higher-level effects of multigenerational Zn exposure in Daphnia magna. Chemosphere. 2010;80(9):1014–20. 20580408 10.1016/j.chemosphere.2010.05.032

[107] Szaflarski W, Sowiński M, Leśniczak M, Ojha S, Aulas A, Dave D, et al. A novel stress response pathway regulates rRNA biogenesis. bioRxiv. 2020;2020.08.16.250183. 10.1101/2020.08.16.250183.

[108] Ray SD, Kamendulis LM, Gurule MW, Yorkin RD, Corcoran GB. Ca2+ antagonists inhibit DNA fragmentation and toxic cell death induced by acetaminophen. Faseb j. 1993;7(5):453–63. 8462787 10.1096/fasebj.7.5.8462787

