# Supplementary material for: Exploring the Sublethal Impacts of Cu and Zn on Daphnia magna: a transcriptomic perspective
Source: BMC Genomics. 2024 Aug 19;25:790. doi: 10.1186/s12864-024-10701-8 (PMC11331620; doi:10.1186/s12864-024-10701-8)
Supplement: Supplementary file 1 — Supplementary Material 1 [file 12864_2024_10701_MOESM1_ESM.docx]

**Supplementary Tables**

**Exploring the Sublethal Impacts of Cu and Zn on *Daphnia magna*: A Transcriptomic Perspective**

Berkay Paylar^1^, Yared H. Bezabhe^1^, Jana Jass^1^, Per-Erik Olsson^1^

^1^Biology, The Life Science Center, School of Science and Technology, Örebro University, Örebro, Sweden.

Table S1. RNA concentration and quality of transcriptomic samples

| Sample Name^#^ | Concentration (ng/uL) | A260 | 260/280 | 260/280 Alert | 260/230 | 260/230 Alert |
| --- | --- | --- | --- | --- | --- | --- |
| Con1 | 97,93 | 2,45 | 1,96 | Met criteria | 2,25 | Met criteria |
| Con2 | 102,53 | 2,56 | 2 | Met criteria | 2,29 | Met criteria |
| Con3 | 100,91 | 2,52 | 2,02 | Met criteria | 2,24 | Met criteria |
| Con4 | 102,47 | 2,56 | 1,96 | Met criteria | 2,2 | Met criteria |
| Zn1 | 95,40 | 2,39 | 1,99 | Met criteria | 2,21 | Met criteria |
| Zn2 | 99,34 | 2,48 | 1,99 | Met criteria | 2,21 | Met criteria |
| Zn3 | 95,36 | 2,38 | 1,99 | Met criteria | 2,15 | Met criteria |
| Zn4 | 96,61 | 2,42 | 1,98 | Met criteria | 2,13 | Met criteria |
| Cu1 | 97,20 | 2,43 | 1,99 | Met criteria | 2,22 | Met criteria |
| Cu2 | 90,70 | 2,27 | 1,99 | Met criteria | 2,16 | Met criteria |
| Cu3 | 99,51 | 2,49 | 1,97 | Met criteria | 2,17 | Met criteria |
| Cu4 | 96,16 | 2,40 | 1,99 | Met criteria | 2,11 | Met criteria |

^#^ Con-Control, Zn-Zinc, Cu-Copper

Table S2: Primer sequences of genes used for qPCR validation of transcriptomics analysis

| **Primer** | **Orientation** | **Sequence (5'->3')** |
| --- | --- | --- |
| *mthfr* | F | AGAGAAGGTGGATGCAGGAG |
|  | R | CGGAGTGAATCGTACGACTG |
| *tsl* | F | GCCACTCCGAATCAATACCC |
|  | R | CATGCGCAATTAAGGATTCTGT |
| *mep* | F | AGCTGGACAGGGATGAAGTT |
|  | R | TAGTAAATCACCGCCCACCA |
| *clca2* | F | TCCAGGACGCTGAAGTTCAA |
|  | R | GCCCATTCCTTCACGAACAC |
| *enpp4* | F | CACGCGACGGAGATGGTC |
|  | R | GCTTGACCGAACGTGAATGT |
| *znt1* | F | TCAGTCAAGATGTCACCAAAGA |
|  | R | GCCAACACAGCCAACAATGA |
| *mt-a* | F | ACGTCTGTTGCCAAAACAATTG |
|  | R | CAACAGGACGTCTTGCATCC |
| *mt-b* | F | TGGAACCGAATGCAAATGCG |
|  | R | CGGACTTGCATGGACAACTG |
| *mt-c* | F | AAAGTGTGCCCTCGTTGTCA |
|  | R | CTTACAGTCGTCCCCACACG |
